# Supplementary material for: Germline T cell receptor exchange results in physiological T cell development and function
Source: Nat Commun. 2023 Feb 1;14:528. doi: 10.1038/s41467-023-36180-1 (PMC9892040; doi:10.1038/s41467-023-36180-1)
Supplement: Supplementary file 1 — Supplementary Information [file 41467_2023_36180_MOESM1_ESM.pdf]

Supplementary Information File

Germline T cell receptor exchange results in physiological T cell development and function

Meagan R. Rollins, Jackson F. Raynor, Ebony A. Miller, Jonah Z. Butler, Ellen J. Spartz, Walker S. Lahr, Adam L. Burrack, Yun You, Branden S. Moriarity, Beau R. Webber, and Ingunn M. Stromnes

Supplementary Figure 1

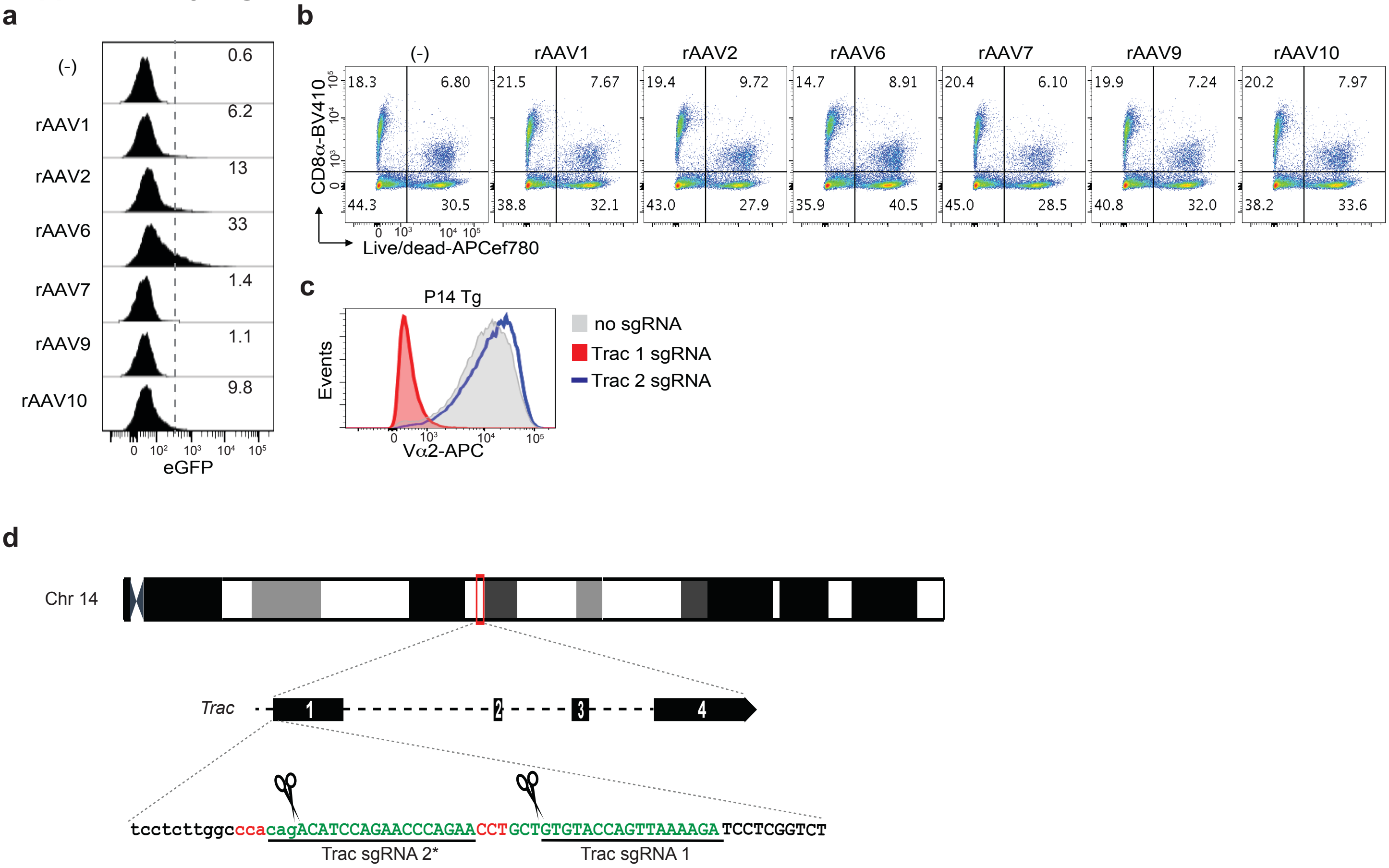

Supplementary Figure 1. Characterization of rAAV serotype infection efficiency and *Trac* sgRNAs in primary activated murine T cells. a rAAV-eGFP serotype infection efficiency in primary activated murine CD8 T cells. Representative of 2 independent experiments. **b** Representative plots showing viability of rAAV-GFP infected T cells on day 3 post rAAV infection, which is day 5 post T cell activation. Representative of 2 independent experiments. **c** Vα2 expression in activated P14 T cells on day 3 post electroporation with *Trac*1 or *Trac*2 sgRNAs complexed to Cas9 RNP. *Trac* 2 sgRNA failed to interfere with transgenic TCR expression indicating it cuts outside of *Trac*. **d** Representation of *Trac1* and *Trac2* sgRNAs on murine chromosome 14.

Supplementary Figure 2

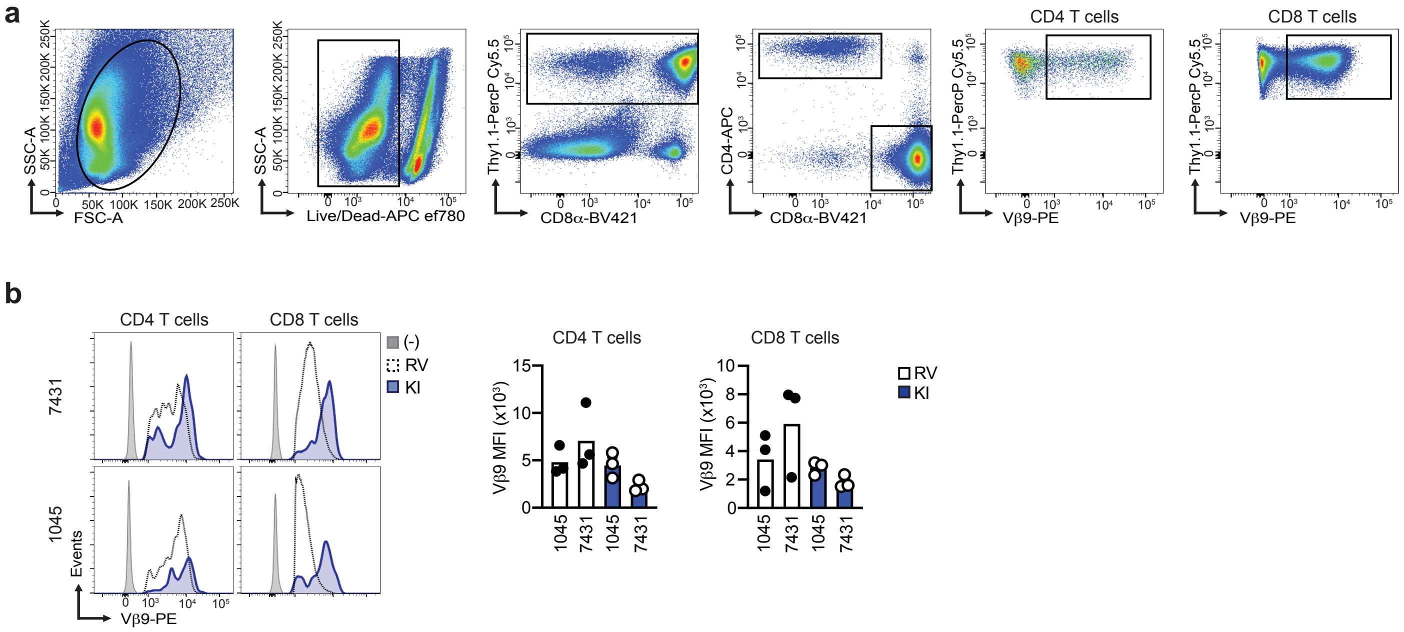

Supplementary Figure 2. Comparison of retrovirally transduced (RV) T cells to CRISPR/Cas9 + rAAV6 *Trac*-targeted TCR knock-in T cells (KI). a Representative gating strategy for analysis TCR engineered T cells. **b** Representative histogram overlays (left) and mean fluorescence intensity (MFI, right graphs) following the RV or KI approaches. MFI is gated on CD4+Vβ9+ or CD8+Vβ9+ T cells. Data are mean ± S.E.M. and are pooled from 2 independent experiments.

Supplementary Figure 3

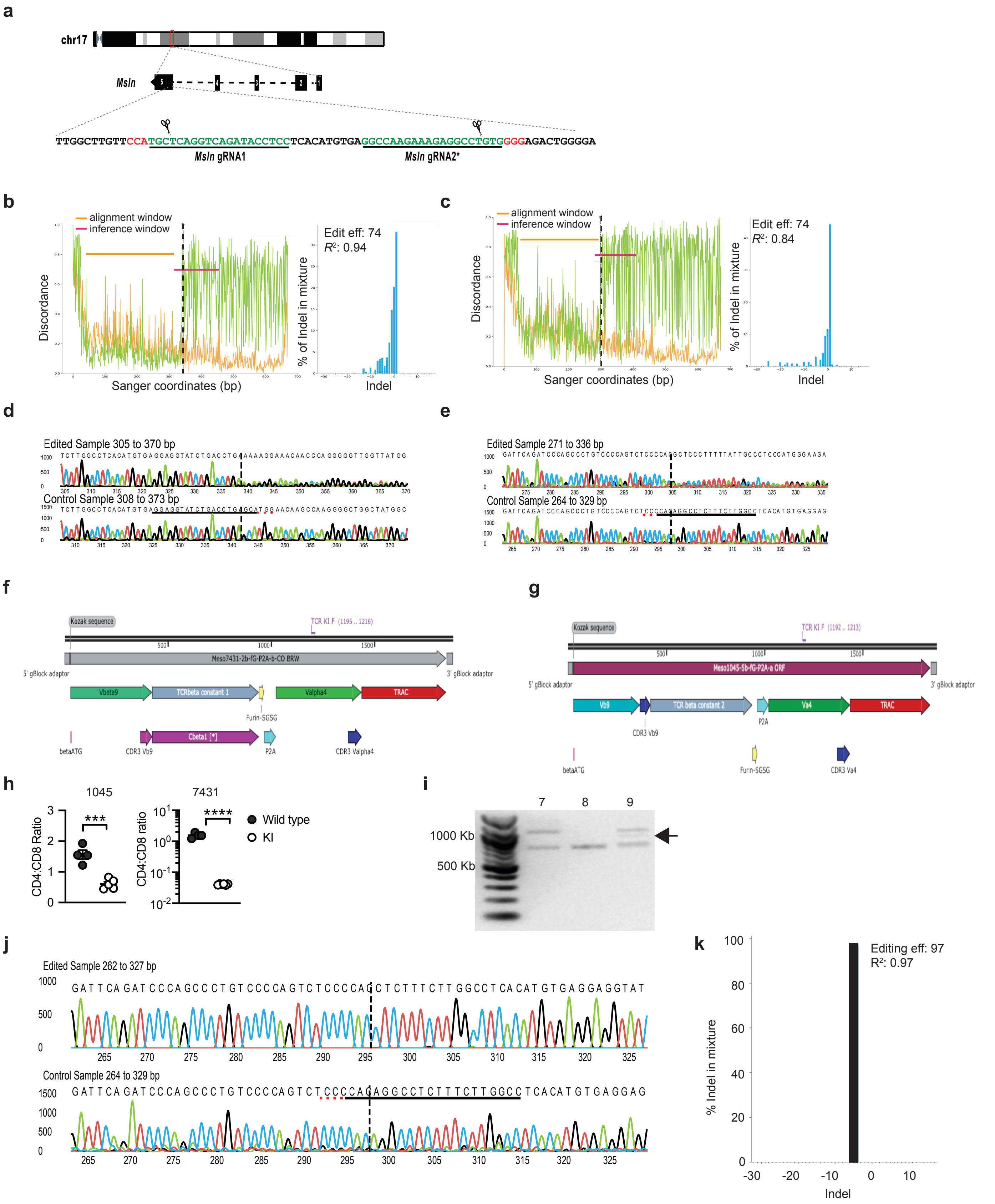

Supplementary Figure 3. Generation of low and high affinity Msln-specific TRex mice that lack *Msln*. a Simplified schematic of the 2 Msln-specific sgRNAs tested. Sanger sequencing and ICE analysis of 3T3 cells electroporated with *Msln* sgRNA #1 (**b**) or *Msln* sgRNA #2 (**c**) complexed with Cas9 RNP. Editing efficiency was determined by ICE analysis (https://ice.synthego.com/#/). Representative sequences of 3T3 cells that were nucleofected with Cas9 RNP complexed with Msln sgRNA #1 (**d**) or sgRNA #2 (**e**). Genetic maps of the 7431 TCR (**f**) and 1045 TCR (**g**). **h** CD4:CD8 T cell ratio in the blood of 1045 and 7431 TRex F0 pups. n=4-6 biologically independent animals. **i** Magnified junction PCR results from 7431 TRex mice (#7-#9) showing an unexpected band in mouse #9 (see Fig. 3i). **j** Representative *Msln* exon 4 sequence from a $7431^{+/+}$*Msln*$^{-/-}$ TRex mouse. **k** Percent indels in *Msln* exon 4 from j.

Supplementary Figure 4

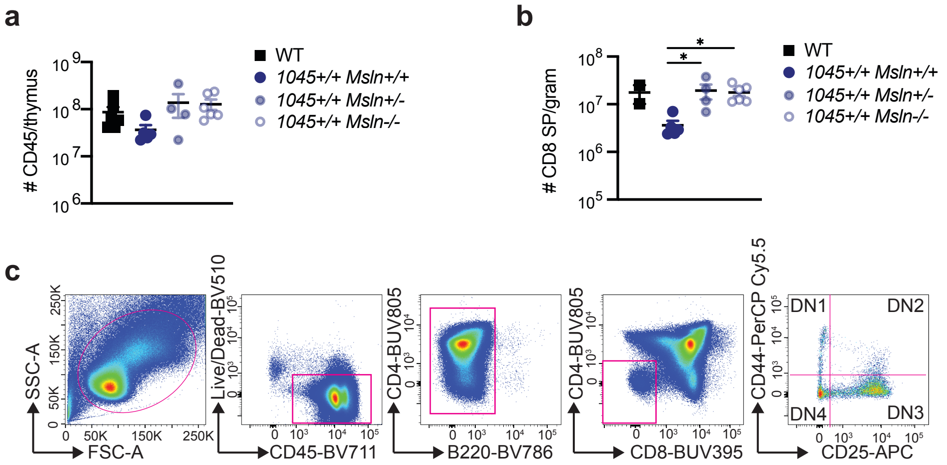

Supplementary Figure 4. Thymocyte development in 1045 TRex mice with decreasing *Msln*. a Number of CD45+ cells per thymus. Data are mean ± S.E.M. n=4-6 biologically independent animals. **b** Number of CD8 SPs per thymus gram. Data are mean ± S.E.M. *p<0.05. One-way ANOVA with a Tukey's posttest was performed among the three 1045 cohorts which included n=4-6 biologically independent animals. WT, n=2 biologically independent animals. **c** Representative gating strategy for DN stage analysis. Note that the doublet exclusion gate is not shown.

Supplementary Figure 5

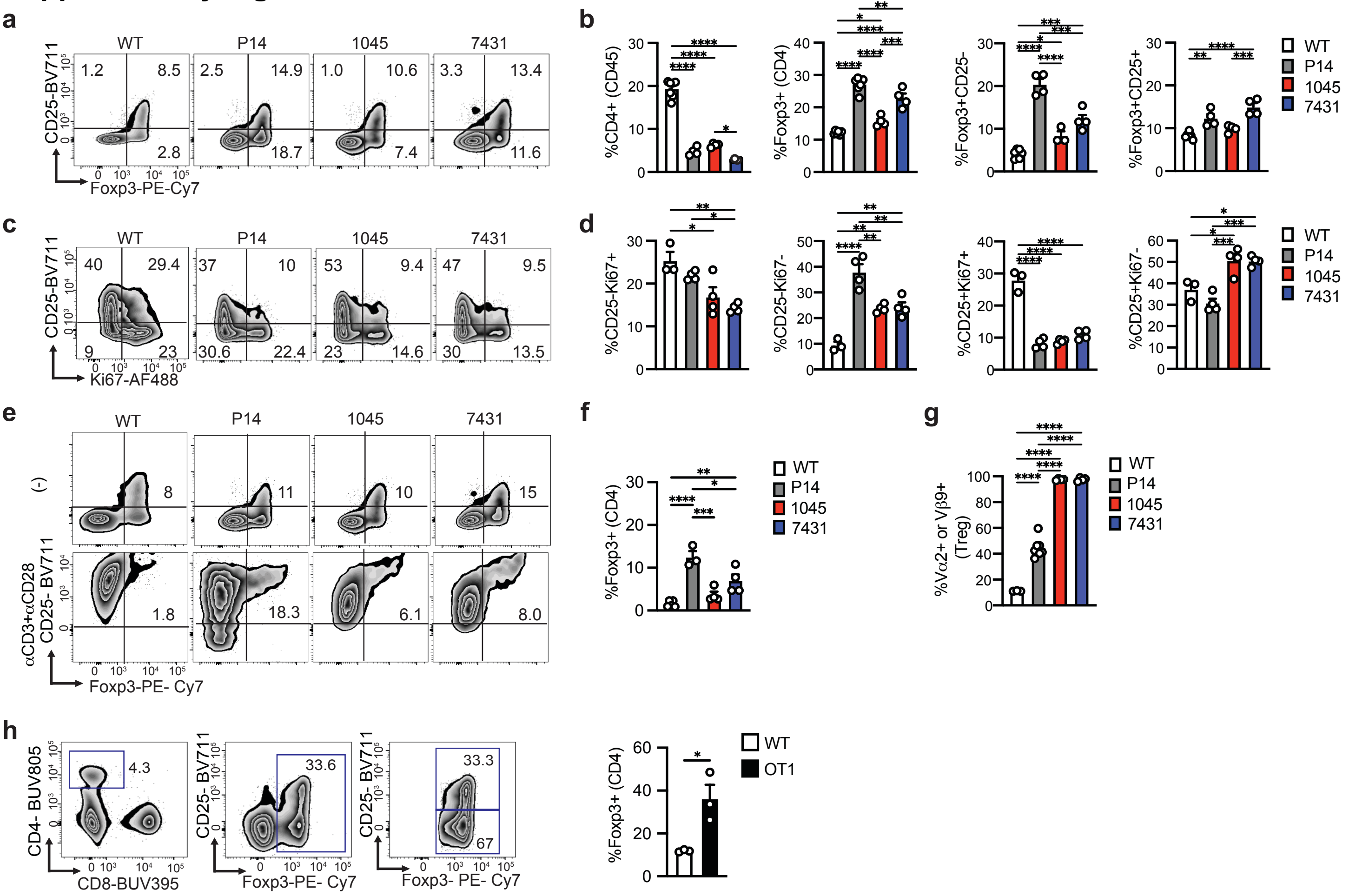

Supplementary Figure 5. Bias toward Tregs in historical MHC class I TCR transgenic mice is obviated in TRex mice. 1045 and 7431 TRex mice are *Msln-/-* in all panels. **a** Representative plots gated on live CD4+CD3+ T cells. **b** Quantified data from a. Data are mean ± S.E.M. n=4-7 biologically independent animals per group. **p*<0.05, ***p*<0.005, ****p*<0.0005, and *****p*<0.0001. One-way ANOVA with a Tukey's posttest. **c** Representative Ki67 and CD25 plots are gated on live CD4+Foxp3+ splenic T cells. **d** Quantified data from c, gated on Foxp3+ Treg. Data are mean ± S.E.M. n=3-4 biologically independent animals per group. **p*<0.05, ***p*<0.005,****p*<0.0005, and *****p*<0.0001. One-way ANOVA with a Tukey's posttest**. e** Representative flow plots gated on CD4+ T cells on day 6 ± *in vitro* activation. **f** Quantification of e. Data are mean ± S.E.M. n=3-5 biologically independent animals per group. **p*<0.05, ***p*<0.005, and ****p*<0.0005. One-way ANOVA with a Tukey's posttest. **g** Proportion of CD4+Foxp3+ T cells that express Vα2 (binds P14 TCR) or Vβ9 (binds 1045 and 7431 TCR) on day 6 post activation. n=3-7 biologically independent animals. One-way ANOVA with a Tukey's posttest**.** *****p*<0.0001. **h** Gating strategy and frequency of Foxp3+ Tregs from representative OT1 TCR transgenic spleen and quantified data (right). Data are mean ± S.E.M. n=3 biologically independent animals per group. **p*<0.05. Unpaired two-tailed Student's t test.

Supplementary Figure 6

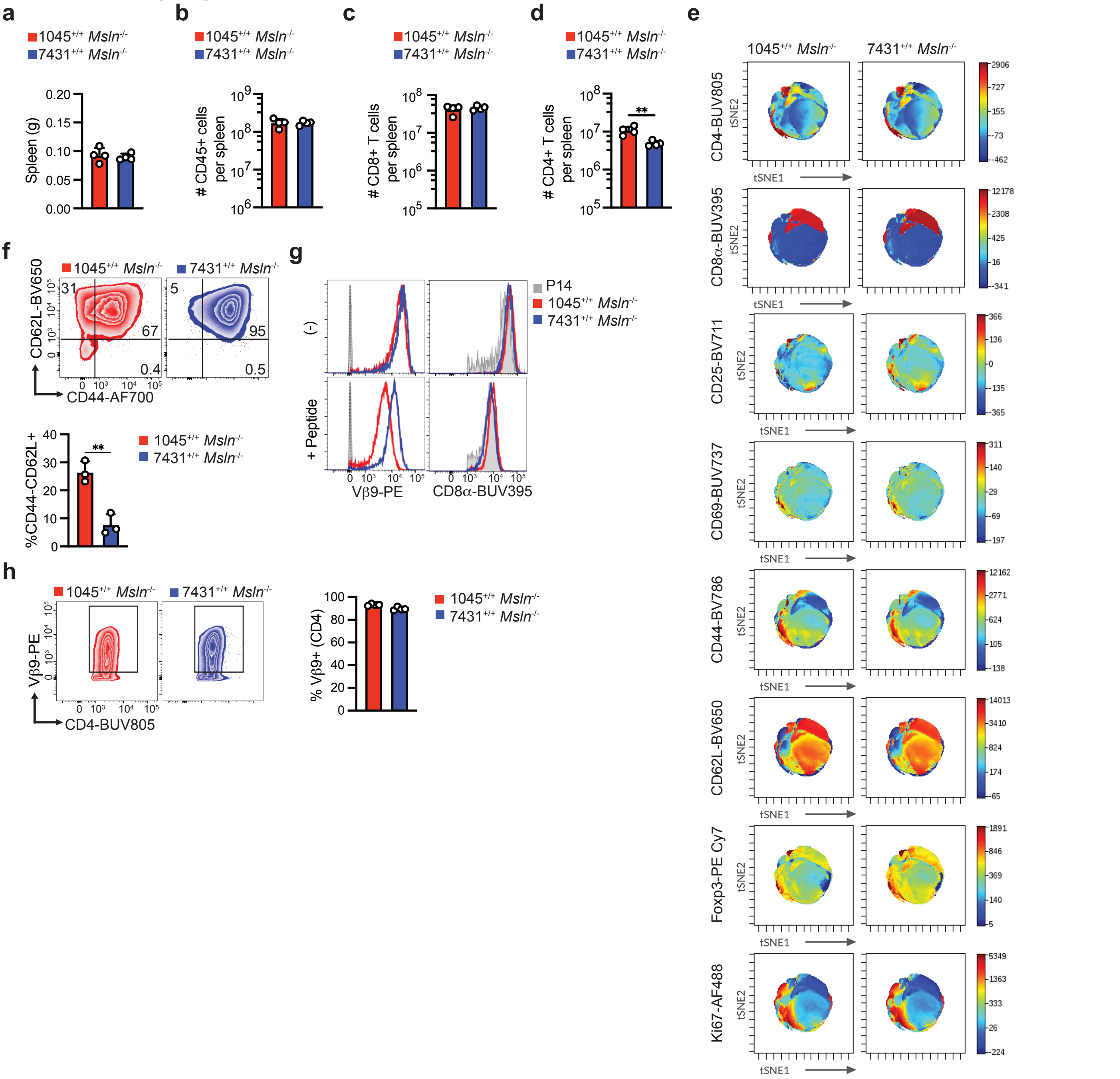

Supplementary Figure 6. Comparison of low and high affinity T cells from TRex *Msln*$^{-/-}$ mice. a Spleen weight in grams (g). Data are mean ± S.E.M. n=4 biologically independent animals per group. **b** Number of CD45+ immune cells per spleen. Data are mean ± S.E.M. n=4 biologically independent animals. **c** Number of CD8+ T cells per spleen. Data are mean ± S.E.M. n=4 biologically independent animals. **d** Number of CD4+ T cells per spleen. Data are mean ± S.E.M. n=4 biologically independent animals. ***p*<0.005. Unpaired two-tailed Student's t test. **e** ViSNE analysis of total CD45+ splenocytes was generated using Cytobank. **f** Representative CD44 and CD62L staining on day 6 post *in vitro* activation with Msln406-414 peptide and recombinant IL-2. Data are quantified below as mean ± S.E.M. n=3 biologically independent animals. ***p*<0.005. Unpaired two-tailed Student's t test. **g** Representative Vβ9 and CD8α staining gated on *in vitro* expanded effector T cells. P14 Tg T cells were treated identically to TRex T cells and used as a negative control for Vβ9 staining. **h** Representative plots gated on CD4 T cells (left) and proportion of CD4 T cells that express Vβ9. Data are mean ± S.E.M. n=4 biologically independent animals.

Supplementary Figure 7

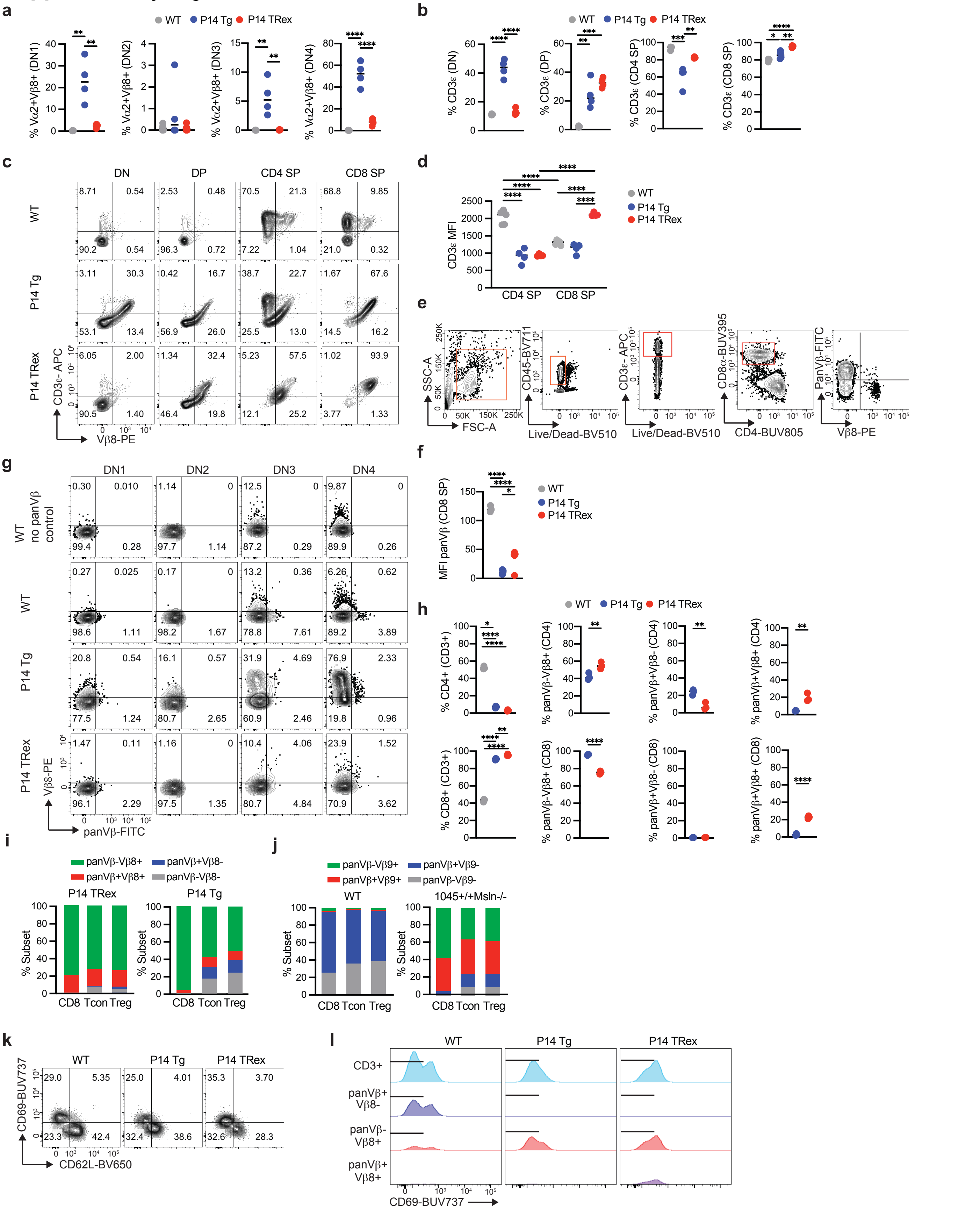

Supplementary Figure 7. Thymocyte maturation in P14 TRex mice. a Frequency of thymocytes in DN1, DN2, DN3 or DN4 that express Vα2 and Vβ8. n=4 biologically independent animals. ** p<0.005, and **** p<0.0001. One-way ANOVA with a Tukey's posttest. **b** Frequency of DN1, DN2, DN3 or DN4 thymocytes that express CD3ε. n=4 biologically independent animals. * p<0.05, ** p<0.005, *** p<0.0005, and **** p<0.0001. One-way ANOVA with a Tukey's posttest. **c** Representative CD3ε and Vβ8 staining among the indicated thymocyte developmental stage. **d** CD3ε mean fluorescence intensity (MFI) of CD4 or CD8 SP thymocytes. n=4-5 biologically independent animals. **** p<0.0001. One-way ANOVA with a Tukey's posttest. **e** Simplified gating strategy for detecting dual TCR Vβ

expressing cells. Note that an additional gate for excluding doubles is not shown. **f** PanVβ MFI of CD8 SP thymocytes. n=4 biologically independent animals. *$p<0.05$ and ****$p<0.0001$. One-way ANOVA with a Tukey's posttest**. g** Representative Vβ8 and panVβ staining gated on thymocyte DN1-DN4 stages. **h** Frequency of CD4 (top row) or CD8 (bottom row) T cells in peripheral blood that express the exogenous (Vβ8) and/or endogenous Vβ. n=3 biologically independent animals. *$p<0.05$, **$p<0.005$, and ****$p<0.0001$. One-way ANOVA with a Tukey's posttest**. i** Mean proportion of circulating CD8 T cells, CD4 Foxp3- conventional T cells (Tcon) or CD4+Foxp3+ Treg that express a single or multiple Vβ from P14 TRex or P14 transgenic (Tg) mice. Data are mean of n=3 biologically independent animals per group. **j** Mean proportion of circulating CD8 T cells, CD4+Foxp3- conventional T cells (Tcon) or CD4+Foxp3+ Treg that express a single or multiple Vβ from wild type (WT) or $1045^{+/+}$*Msln*$^{-/-}$ TRex mice. n=3 mice per group. **k** Representative CD69 and CD62L staining among CD3+CD8+SP thymocytes. **l** Representative CD69 staining gated on the indicated CD3+CD8+SP thymocyte subset.

Supplementary Figure 8

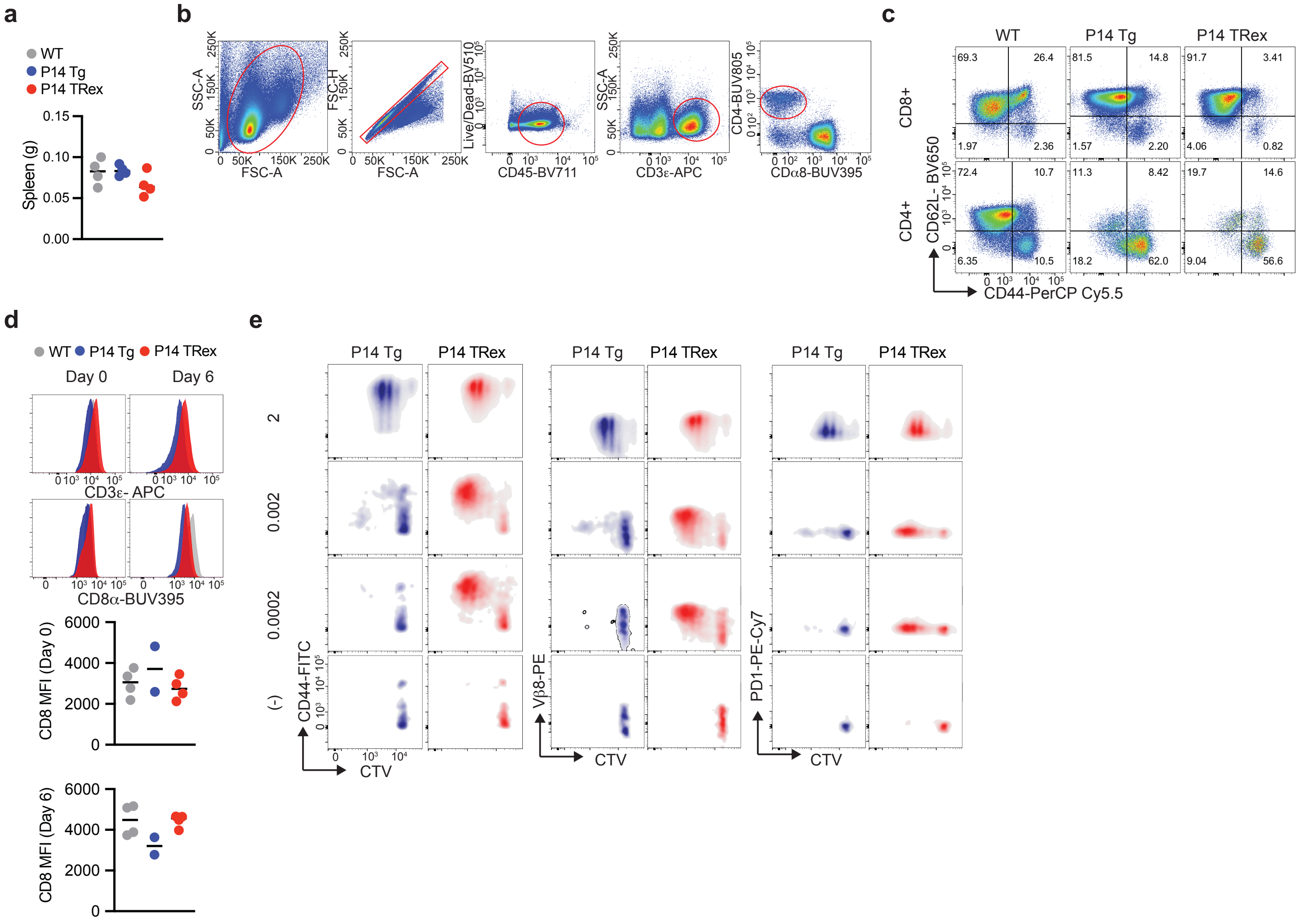

Supplementary Figure 8. Targeting the P14 TCR to *Trac* enhances antigen sensitivity. a Spleen weight in grams (g). Data are mean ± S.E.M. n=4 mice per group. **b** Representative gating schematic for splenic T cell analysis. **c** CD62L and CD44 staining to distinguish naïve/effector/memory phenotype gated on CD8 or CD4 T cells. **d** Histograms gated on CD8 T cells either ex vivo (day 0) or 6 days post activation. CD8α MFI at both timepoints is quantified below. n=2-4 biologically independent animals per group. **e** Proliferation and phenotype gated on CD8 T cells 3 days post activation with gp33 peptide without exogenous IL-2.

Supplementary Table 1. Genomic and flow cytometric analysis of TCR expression in P14 TRex mice

#	ID	Sex	P14[&]	%CD8[Φ]	%Vβ8 (CD8)	%CD4	%Vβ8 (CD4)
1	7043	F	-/-	n.d.	n.d.	n.d.	n.d.
2	7044	F	-/-	n.d.	n.d.	n.d.	n.d.
3	7045	F	-/-	n.d.	n.d.	n.d.	n.d.
4	7046	F	-/-	n.d.	n.d.	n.d.	n.d.
5	7047	F	+/-	29.6	99.2	1.15	48.6
6	7048	M	-/-	n.d.	n.d.	n.d.	n.d.
7	7049	F	-/-	n.d.	n.d.	n.d.	n.d.
8	7050	F	+/-	31.3	99.5	0.74	52.3
9	7051	F	-/-	n.d.	n.d.	n.d.	n.d.
10	7052	F	-/-	n.d.	n.d.	n.d.	n.d.
11	7053	M	-/-	11.7	6.27	19.2	5.07
12	7054	M	-/-	n.d.	n.d.	n.d.	n.d.
13	7055	M	-/-	1.19	1.9	1.7	1.07
14	7056	M	-/-	n.d.	n.d.	n.d.	n.d.
15	7057	F	-/-	n.d.	n.d.	n.d.	n.d.
16	7058	F	-/-	n.d.	n.d.	n.d.	n.d.
17	7059	F	+/-	n.d.	n.d.	n.d.	n.d.
18	7060	F	n.r.	11	6.97	16.4	4.83
19	7061	F	-/-	n.d.	n.d.	n.d.	n.d.
20	7062	M	+/-	30.1	91.7	6.32	21
21	7063	M	-/-	10.5	9.39	19.2	5.68
22	7064	F	-/-	n.d.	n.d.	n.d.	n.d.
23	7065	F	-/-	n.d.	n.d.	n.d.	n.d.
24	7066	F	-/-	n.d.	n.d.	n.d.	n.d.
25	7067	F	-/-	n.d.	n.d.	n.d.	n.d.
26	7068	F	+/+	n.d.	n.d.	n.d.	n.d.
27	7069	M	+/-	23.8	98.2	1.03	25.1
28	7070	M	+/+	29.8	95.8	3.72	18.4
29	7071	M	+/+	35	90.9	7.23	8.74
30	7072	M	n.r.	5.68	4.12	4.92	2.38
31	7073	M	+/-	27.1	95.5	2.34	36.1
32	7074	M	n.r.	11.4	7.97	14.5	4.41
33	7075	M	+/+	30.3	95	4.41	6.32
34	7076	M	-/-	n.d.	n.d.	n.d.	n.d.
35	7077	F	-/-	n.d.	n.d.	n.d.	n.d.
36	7078	M	-/-	n.d.	n.d.	n.d.	n.d.
37	7079	M	-/-	n.d.	n.d.	n.d.	n.d.
38	7080	M	+/+	25.1	98.1	0.9	55
39	7081	M	+/+	30.3	96.5	4.77	35.3
40	7082	M	+/-	30.9	96.3	3.36	26.2
41	7083	M	+/-	33	95.4	3.58	5.55
42	7084	F	+/-	26	99.1	0.75	55.3
43	7085	F	+/-	26.2	98.8	1.06	35
44	7086	F	-/-	9.71	7.54	14.4	5.16
45	7087	M	n.r.	23	98.7	1.29	33.2
46	7088	M	n.r.	10	6.54	16.3	4.41
47	7089	M	+/-	18.2	97.9	0.64	47.5
48	7090	M	n.r.	11.1	6.05	15.3	4.27
49	7091	M	+/-	25.7	97.9	1	53.5
50	7092	F	n.r.	11.3	7.6	13.4	4.76
51	7093	M	n.r.	31.1	91.2	6.84	5.97
52	7094	M	+/-	28	81.1	9.9	17.1

[&]P14 TCR *Trac* knock-in was determined by junction PCR of isolated tail DNA from pups; n.r., no results, sequence analysis was attempted but data were inconclusive.[Φ]n.d., not determined

Supplementary Table 2. Summary of zygote engineering using CRISPR/Cas9.

**Virus**	**Total # zygotes**	**# 2-cell embryos**	**# 1-cell embryos**	**# Lysed zygotes**	**# Pseudopregnant CD-1 females**	**# Pups**
1045	91	61	27	3	2	15
7431	256	206	43	7	4	13
P14	471	235	226	10	8	52

Supplementary Table 3. Antibodies used for flow cytometry and *in vitro* T cell activation.

Target	Fluorophore	Clone	Vendor	Cat no.	Lot #	Dilution
CD16/32	Purified	2.4G2	Tonbo Biosciences	70-0161-U500	D0161070920704	1:100
CD28 (for T cell activation)	Purified	37.51	BD Biosciences	553294	1039453	1:1000
CD3ε (for T cell activation)	Purified	145-2C11	BD Biosciences	553057	1307189	1:1000
CD24	FITC	M1/69	BD Biosciences	553261	8071818	1:100
CD25	BV711	PC61	BioLegend	102049	B359380	1:100
CD25	APC	PC61	BioLegend	102012	1284406	1:100
CD3ε	BV650	145-2C11	BD Biosciences	564378	B350667	1:100
CD3ε	PE-Cy7	145-2C11	BD Biosciences	56110	2489229	1:100
CD3ε	APC	145-2C11	Tonbo Biosciences	20-0031-U100	C0031081215203	1:100
CD4	APC	RM4-5	BD Biosciences	557681	7045882	1:100
CD4	BUV805	GK1.5	BD Biosciences	612900	1134156	1:100
CD44	PerCP/Cy5.5	IM7	BioLegend	103032	B333710	1:100
CD44	AF700	IM7	BioLegend	103026	B345604	1:100
CD44	BV786	IM7	BioLegend	103059	B357479	1:100
CD44	FITC	IM7	BioLegend	103006	B156153	1:100
CD45	BV711	30-F11	BD Biosciences	563709	1076257	1:100
CD45.1	APC/Cy7	A20	eBiosciences	47-0453-82	E10196-1636	1:100
CD45R/B220	BV786	RA3-6B2	BD Biosciences	563894	8250698	1:100
CD62L	BV650	MEL-14	BioLegend	104453	B324616	1:100
CD69	BUV737	H1.2F3	BD Biosciences	612793	1159821	1:100
CD8α	BUV395	53-6.7	BD Biosciences	563786	1207296	1:100
CD8α	BV421	53-6.7	BioLegend	100737	B284315	1:100
FoxP3	PE/Cy7	FJK-16s	Invitrogen	25-577382	2254250	1:75
IFNγ	APC	B27	BD Biosciences	554702	4031676	1:100
Ki67	AF488	B56	BD Biosciences	561165	1133448	1:75
Live/Dead	APC ef780	N/A	Tonbo Biosciences	13-0865-T100	D0865031422133	1:500
Live/Dead	BV510	N/A	Tonbo Biosciences	13-0870-T100	D0870111920133	1:1000
PD-1	PE-Cy7	J43	eBiosciences	25-9985-82	E15003-107	1:100
TCR βchain	BUV737	H57-597	BD Biosciences	564799	1069239	1:100
TCR Vβ8.1/8.2	PE	KJ16	eBiosciences	12-5813-80	E028050	1:100
TCR Vβ9	PE	MR10-2	BioLegend	139804	B252004	1:100
Thy1.1	PerCP/Cy5.5	Ox-7	BioLegend	202516	B234918	1:100
TNFα	BV711	MP6-XT22	BioLegend	506349	B361002	1:100
Vα2 TCR	APC	B20.1	BD Biosciences	560622	1308849	1:200
**BD Mouse Vβ TCR screening panel KIT**	FITC		BD Biosciences	557004	2140903	Pre-diluted
Vβ2 TCR (from screening kit)	FITC	B20.6	BD Biosciences	51-01634L	2098555	Pre-diluted
Vβ3 TCR (from screening kit)	FITC	KJ25	BD Biosciences	51-01404L	2098551	Pre-diluted
Vβ4 TCR (from screening kit)	FITC	KT4	BD Biosciences	51-01934L	2098568	Pre-diluted
Vβ5.1/5.2 TCR (from screening kit)	FITC	MR9-4	BD Biosciences	51-01354L	2098546	Pre-diluted
Vβ6 TCR (from screening kit)	FITC	RR4-7	BD Biosciences	51-01364L	2098547	Pre-diluted
Vβ7 TCR (from screening kit)	FITC	TR310	BD Biosciences	51-01424L	2098553	Pre-diluted
Vβ9 TCR (from screening kit)	FITC	MR10-2	BD Biosciences	51-01384L	2098549	Pre-diluted
Vβ10b TCR (from screening kit)	FITC	B21.5	BD Biosciences	51-01644L	2098556	Pre-diluted
Vβ11 TCR (from screening kit)	FITC	RR3-15	BD Biosciences	51-01374L	2098548	Pre-diluted
Vβ12 TCR (from screening kit)	FITC	MR11-1	BD Biosciences	51-01684L	2098557	Pre-diluted
Vβ13 TCR (from screening kit)	FITC	MR12-3	BD Biosciences	51-01394L	2098550	Pre-diluted
Vβ14 TCR (from screening kit)	FITC	14-2	BD Biosciences	51-01564L	2098554	Pre-diluted
Vβ17a TCR (from screening kit)	FITC	KJ23	BD Biosciences	51-01414L	2098552	Pre-diluted
