# Supplementary material for: Germline T cell receptor exchange results in physiological T cell development and function
Source: Nat Commun. 2023 Feb 1;14:528. doi: 10.1038/s41467-023-36180-1 (PMC9892040; doi:10.1038/s41467-023-36180-1)
Supplement: Supplementary file 3 — Reporting Summary [file 41467_2023_36180_MOESM3_ESM.pdf]

nature portfolio

Corresponding author(s): Ingunn M. Stromnes

Last updated by author(s): Dec 16, 2022

Reporting Summary

Nature Portfolio wishes to improve the reproducibility of the work that we publish. This form provides structure for consistency and transparency in reporting. For further information on Nature Portfolio policies, see our Editorial Policies and the Editorial Policy Checklist.

Statistics

For all statistical analyses, confirm that the following items are present in the figure legend, table legend, main text, or Methods section.

n/a	Confirmed	
☐	☒	The exact sample size (*n*) for each experimental group/condition, given as a discrete number and unit of measurement
☐	☒	A statement on whether measurements were taken from distinct samples or whether the same sample was measured repeatedly
☐	☒	The statistical test(s) used AND whether they are one- or two-sided<br>*Only common tests should be described solely by name; describe more complex techniques in the Methods section.*
☒	☐	A description of all covariates tested
☐	☒	A description of any assumptions or corrections, such as tests of normality and adjustment for multiple comparisons
☐	☒	A full description of the statistical parameters including central tendency (e.g. means) or other basic estimates (e.g. regression coefficient) AND variation (e.g. standard deviation) or associated estimates of uncertainty (e.g. confidence intervals)
☐	☒	For null hypothesis testing, the test statistic (e.g. *F*, *t*, *r*) with confidence intervals, effect sizes, degrees of freedom and *P* value noted<br>*Give P values as exact values whenever suitable.*
☒	☐	For Bayesian analysis, information on the choice of priors and Markov chain Monte Carlo settings
☒	☐	For hierarchical and complex designs, identification of the appropriate level for tests and full reporting of outcomes
☒	☐	Estimates of effect sizes (e.g. Cohen's *d*, Pearson's *r*), indicating how they were calculated

Our web collection on statistics for biologists contains articles on many of the points above.

Software and code

Policy information about availability of computer code

Data collection	Data were acquired using FACs Diva (version 8.0.1) for flow cytometry acquisition. Images were acquired using Imaris 9.1.0 (Bitplane).
Data analysis	Flow cytometry data was analyzed using Flow Jo v10.7.1. Data were exported and compiled in Microsoft Excel (v16.15.1) and statistical significance and generation of graphs used Graphpad Prism (v9.0). Cytobank (version 10) was used for ViSNE analysis of flow cytometry data. Snapgene (v5.2.5) and ICE analysis software (V3.0) was used to analyze genomic edits of the DNA as done by CRISPR/Cas9.

For manuscripts utilizing custom algorithms or software that are central to the research but not yet described in published literature, software must be made available to editors and reviewers. We strongly encourage code deposition in a community repository (e.g. GitHub). See the Nature Portfolio guidelines for submitting code & software for further information.

Data

Policy information about availability of data

All manuscripts must include a data availability statement. This statement should provide the following information, where applicable:

- Accession codes, unique identifiers, or web links for publicly available datasets
- A description of any restrictions on data availability
- For clinical datasets or third party data, please ensure that the statement adheres to our policy

Data are available in a Source Data file, Article, or Supplementary Information. All data are available from the corresponding author upon reasonable request.

Human research participants

Policy information about studies involving human research participants and Sex and Gender in Research.

Reporting on sex and gender	n/a
Population characteristics	n/a
Recruitment	n/a
Ethics oversight	n/a

Note that full information on the approval of the study protocol must also be provided in the manuscript.

Field-specific reporting

Please select the one below that is the best fit for your research. If you are not sure, read the appropriate sections before making your selection.

☒ Life sciences ☐ Behavioural & social sciences ☐ Ecological, evolutionary & environmental sciences

For a reference copy of the document with all sections, see nature.com/documents/nr-reporting-summary-flat.pdf

Life sciences study design

All studies must disclose on these points even when the disclosure is negative.

Sample size	Statistical analysis was carried out using GraphPad Prism Software (v9.0). Statistical analysis was conducted on data from three or more biologically independent experimental replicates. For power of 80%, the level of significance was set at 5%, 3-6 mice in each group was estimated. Data are a combination of a minimum of 2 independent experiments. Data distribution was assumed to be normal, but this was not formally tested. Comparisons between groups were planned before statistical testing and target effect sizes were not predetermined. Appropriate statistical methods were used to calculate significance as described in the figure legends. Unpaired, two-tailed student's T test was used to compare 2-group data unless otherwise indicated. One-way analysis of variance (ANOVA) and Tukey post-test were used for >2-group data. In Figure 8 panels e, f, and i, significance was determined by a multiple Unpaired T-test with Welch correction a false discovery rate (FDR) of 1% and a two-stage step-up (see reference 73). Graphed data are presented as mean ± standard error of the mean (S.E.M.) unless otherwise indicated and p<0.05 was considered significant. *p<0.05, **p<0.005, ***p<0.0005, and ****p<0.0001.
Data exclusions	No data was excluded.
Replication	The number of independent experimental replicates is given in each figure legend, typically reflecting 1 to 3 repeat experiments. All reported attempts at replication were successful.
Randomization	Mice were enrolled into the experiments based off of genotype for TCR knock-in and msln. No randomization method was used.
Blinding	Data collection and Analysis was not performed blind.

Reporting for specific materials, systems and methods

We require information from authors about some types of materials, experimental systems and methods used in many studies. Here, indicate whether each material, system or method listed is relevant to your study. If you are not sure if a list item applies to your research, read the appropriate section before selecting a response.

Materials & experimental systems

n/a	Involved in the study	
☐	☒	Antibodies
☐	☒	Eukaryotic cell lines
☒	☐	Palaeontology and archaeology
☐	☒	Animals and other organisms
☒	☐	Clinical data
☒	☐	Dual use research of concern

Methods

n/a	Involved in the study	
☒	☐	ChIP-seq
☐	☒	Flow cytometry
☒	☐	MRI-based neuroimaging

Antibodies

Antibodies used

CD24 FITC clone: M1/69 BD Biosciences Cat# 553261 Lot# 8071818 Dilution 1:100
CD25 BV711 clone: PC61 BioLegend Cat# 102049 Lot# B359380 Dilution 1:100
CD25 APC clone: PC61 BioLegend Cat# 102012 Lot# 1284406 Dilution 1:100
CD3e BV650 clone: 145-2C11 BD Biosciences Cat# 564378 Lot# B350667 Dilution 1:100
CD3e PE-Cy7 clone: 145-2C11 BD Biosciences Cat# 56110 Lot# 2489229 Dilution 1:100
CD3e APC clone: 145-2C11 Tonbo Biosciences Cat# 20-0031-U100 Lot# C0031081215203 Dilution 1:100
CD4 APC clone: RM4-5 BD Biosciences 5 Cat# 57681 Lot# 7045882 Dilution 1:100
CD4 BUV805 clone: GK1.5 BD Biosciences Cat# 612900 Lot# 1134156 Dilution 1:100
CD44 PerCP/Cy5.5 clone: IM7 BioLegend Cat# 103032 Lot# B333710 Dilution 1:100
CD44 AF700 clone: IM7 BioLegend Cat# 103026 Lot# B345604 Dilution 1:100
CD44 BV786 clone: IM7 BioLegend Cat# 103059 Lot# B357479 Dilution 1:100
CD44 FITC clone: IM7 BioLegend Cat# 103006 Lot# B156153 Dilution 1:100
CD45 BV711 clone: 30-F11 BD Biosciences Cat# 563709 Lot# 1076257 Dilution 1:100
CD45.1 APC/Cy7 clone: A20 ebiosciences Cat# 47-0453-82 Lot# E10196-1636 Dilution 1:100
CD45R/B220 BV786 clone: RA3-6B2 BD Biosciences Cat# 563894 Lot# 8250698 Dilution 1:100
CD62L BV650 clone: MEL-14 BioLegend Cat# 104453 Lot# B324616 Dilution 1:100
CD69 BUV737 clone: H1.2F3 BD Biosciences Cat# 612793 Lot# 1159821 Dilution 1:100
CD8a BUV395 clone: 53-6.7 BD Biosciences Cat# 563786 Lot# 1207296 Dilution 1:100
CD8a BV421 clone: 53-6.7 BioLegend Cat# 100737 Lot# B284315 Dilution 1:100
FoxP3 PE/Cy7 clone: FJK-16s Invitrogen Cat# 25-577382 Lot# 2254250 Dilution 1:75
IFNy APC clone: B27 BD Biosciences Cat# 554702 Lot# 4031676 Dilution 1:100
Ki67 AF488 clone: B56 BD Biosciences Cat# 561165 Lot# 1133448 Dilution 1:75
Live/Dead APC ef780 clone: N/A Tonbo Biosciences Cat#13-0865-T100 Lot# D0865031422133 Dilution 1:500
Live/Dead BV510 clone: N/A Tonbo Biosciences Cat#13-0870-T100 Lot# D0870111920133 Dilution 1:1000
PD-1 PE-Cy7 clone: J43 ebiosciences Cat# 25-9985-82 Lot# E15003-107 Dilution 1:100
TCR B chain BUV737 clone: H57-597 BD Biosciences Cat# 564799 Lot# 1069239 Dilution 1:100
TCR V beta 8.1/8.2 PE clone: KJ16 eBiosciences Cat# 12-5813-80 Lot# E028050 Dilution 1:100
TCR Vb9PE clone: MR10-2 BioLegend Cat# 139804 Lot# B252004 Dilution 1:100
Thy1.1 PerCP/Cy5.5 clone: Ox-7 BioLegend Cat# 202516 Lot# B234918 Dilution 1:100
TNFa BV711 clone: MP6-XT22 BioLegend Cat# 506349 Lot# B361002 Dilution 1:100
Va2 TCR APC clone: B20.1 BD Biosciences Cat# 560622 Lot# 1308849 Dilution 1:200BD

Mouse Vb TCR screening panel KIT FITC BD Biosciences Cat#557004 Lot#2140903 No dilution was performed as antibodies are supplied pre-diluted. Antibodies used from kit are as follows:
Vb2 TCR FITC clone: B20.6 BD Biosciences Cat# 51-01634L Lot# 2098555 No dilution
Vb3 TCR FITC clone:KJ25 BD Biosciences Cat# 51-01404L Lot# 2098551 No dilution
Vb4 TCR FITC clone:KT4 BD Biosciences Cat# 51-01934L Lot# 2098568 No dilution
Vb5.1/5.2 TCR FITC clone:MR9-4 BD Biosciences Cat# 51-01354L Lot# 2098546 No dilution
Vb6 TCR FITC clone:RR4-7 BD Biosciences Cat# 51-01364L Lot# 2098547 No dilution
Vb7 TCR FITC clone:TR310 BD Biosciences5 Cat# 1-01424L Lot# 2098553 No dilution
Vb9 TCR FITC clone:MR10-2 BD Biosciences Cat# 51-01384L Lot# 2098549 No dilution
Vb10b TCR FITC clone:B21.5 BD Biosciences Cat# 51-01644L Lot# 2098556 No dilution
Vb11 TCR FITC clone:RR3-15 BD Biosciences Cat# 51-01374L Lot# 2098548 No dilution
Vb12 TCR FITC clone:MR11-1 BD Biosciences Cat# 51-01684L Lot# 2098557 No dilution
Vb13 TCR FITC clone:MR12-3 BD Biosciences Cat# 51-01394L Lot# 2098550 No dilution
Vb14 TCR FITC clone:14-2 BD Biosciences Cat# 51-01564L Lot# 2098554 No dilution
Vb17a TCR FITC clone:KJ23 BD Biosciences Cat# 51-01414L Lot# 2098552 No dilution

CD16/32 clone:2.4G2 Tonbo Biosciences Cat# 70-0161-U500 Lot# D0161070920704 Dilution 1:100
CD28, NA/LE, Clone: 37.51 BD Biosciences Cat#553294 Lot# 1039453 1:1000
CD3, NA/LE, Clone: 145 2C11 BD Biosciences Cat# 553057 Lot# 1307189 1:1000

Validation

All antibodies were validated by and purchased from the following commercial vendors as specified by the manufacturer. Antibodies from BioLegend, BD Biosciences, Tonbo Biosciences, eBioscience, and Invitrogen were subject to quality control testing by the manufacturer and validation data including use on mouse cells is available on the vendor website for each product number listed. Antibodies were also validated in-house under the staining conditions used.

Eukaryotic cell lines

Policy information about cell lines and Sex and Gender in Research

Cell line source(s)

EL4 cells are derived from a lymphoma induced in a C57BL/6N mouse by 9,10-dimethyl-1,2-benzanthracene and are commercially available (TIB-93, ATCC). We sorted CD3+ EL4 cells to obtain a pure population for gene engineering. NIH/3T3 fibroblast cell line that was isolated from a mouse NIH/Swiss embryo and are commercially available (CRL-1658, ATCC). Both cell lines were cultured according to ATCC specifications.

Authentication	The cell lines were not authenticated.
Mycoplasma contamination	Cell lines were negative for Mycoplasm.
Commonly misidentified lines (See ICLAC register)	No misidentified cell lines were used.

Animals and other research organisms

Policy information about studies involving animals; ARRIVE guidelines recommended for reporting animal research, and Sex and Gender in Research

Laboratory animals	University of Minnesota Institutional Animal Care and Use Committee approved all animal studies. Mice were co-housed in a SPF facility. Both female and male mice between the ages of 6-12 weeks old were used in this study. C57BL/6J wild type (WT) mice were purchased from Jackson Labs (000664). Pseudopregnant CD-1 female mice were purchased from Charles River Laboratory (CD-1 022). Generation of TRex animals was performed in the Mouse Genetic Laboratory at the University of Minnesota. B6.129P2-B2mtm1Unc/DcrJ (B2m-/-) mice backcrossed to C57Bl/6 strain for 11 generations were purchased from Jackson Labs (002087) and backcrossed to 1045+/+ TRex mice. P14 and OT1 TCR transgenic were kindly provided by Dr. Stephen Jameson and Dr. Vaiva Vezys (University of Minnesota). The vivarium is maintained at a 14:10h light:dark cycle, 68-70F temperature, and 20-70% humidity range.
Wild animals	The study did not involve wild animals.
Reporting on sex	Both female and male mice were used in this study.
Field-collected samples	The study did not involve animals collected from the field.
Ethics oversight	Studies were approved by the Institutional Animal Care and Use Committee (IACUC) at the University of Minnesota.

Note that full information on the approval of the study protocol must also be provided in the manuscript.

Flow Cytometry

Plots

Confirm that:

☒ The axis labels state the marker and fluorochrome used (e.g. CD4-FITC).

☒ The axis scales are clearly visible. Include numbers along axes only for bottom left plot of group (a 'group' is an analysis of identical markers).

☒ All plots are contour plots with outliers or pseudocolor plots.

☒ A numerical value for number of cells or percentage (with statistics) is provided.

Methodology

Sample preparation	Mice were euthanized by cervical dislocation after carbon dioxide sedation. Spleens or thymi were harvested mechanically dissociated to single cells. RBCs (from spleens) were lysed by incubation in 1 mL of Tris-ammonium chloride (ACK) lysis buffer (GIBCO) for 1-2 minutes at room temperature. 9 mL of T cell media was added to quench lysis. Cells were spun at 1400 rpm for 5 minutes at 4° C and resuspended in T cell media and stored on ice until further use. For PBMCs, 100-200 µl of blood was collected per animal in 20 mM EDTA in a 96-well round bottom plate. RBCs were lysed by resuspension in 150 µl ACK lysis buffer (GIBCO) for 10 minutes at room temperature. 1mL of T cell media was added to quench cell lysis. Cells were spun at 350 x g for 5 minutes at 4°C, supernatant decanted. Cells were passed through a 60um nylon mesh and resulting signle cell suspensions were centrifuged and resuspended in a defined volume of FACs buffer, prior to being transfered to a 96-well plate for staining with fluprescent antibody cocktails and MHC-I tetramers.<br>See also Methods section.
Instrument	Cells were acquired with a Fortessa 1770 or Fortessa X-20 with five lasers and using Facs Diva software (BD Biosciences) for running.
Software	Data were analyzed using FlowJo software (version 10) or Cytobank (version 10).
Cell population abundance	CD3+ EL4 cells were sorted to >95% purity. For ex vivo analysis, 50,000-200,000 live cells were analyzed. For in vitro experiments, 20,000-50,000 cells are collected when possible. The proportion of cells is displayed on flow cytometry plots and absolute counts were calculated using counting beads.
Gating strategy	Cells were gated on FSC-A by SSC-A. Single cells were gated by FSC-A by FSC-H. Live CD45+ immune cells by gating on cells that did not stain with the viability stain. From this, different gates were set depending on the desired T cell population.

☒ Tick this box to confirm that a figure exemplifying the gating strategy is provided in the Supplementary Information.
